# Economic evaluation of telemedicine-based integrated management of atrial fibrillation in rural China: a modeling analysis

**DOI:** 10.1038/s41746-026-02772-6

**Published:** 2026-05-19

**Authors:** Wen Liu, Mingfang Li, Yuxuan Cai, Ming Chu, Shimeng Zhang, Gang Yang, Zhihang Peng, Gregory Y. H. Lip, Minglong Chen

**Affiliations:** 1 Department of Biostatistics, School of Public Health, Nanjing Medical University, Nanjing, China; 2 Division of Cardiology, The First Affiliated Hospital with Nanjing Medical University, Nanjing, China; 3 Liverpool Centre for Cardiovascular Science at University of Liverpool, Liverpool John Moores University and Liverpool Heart & Chest Hospital, Liverpool, United Kingdom; 4 Department of Clinical Medicine, Aalborg University, Aalborg, Denmark; 5 Medical University of Bialystok, Bialystok, Poland; 6 Chinese PLA General Hospital, Beijing, China; 7 Jinling Hospital, Medical School of Nanjing University, Nanjing, China; 8 Department of Radiology, The First Affiliated Hospital of Nanjing Medical University, Nanjing, China

**Keywords:** Cardiology, Diseases, Health care, Medical research

## Abstract

Atrial fibrillation (AF) imposes a substantial disease and economic burden worldwide, particularly in rural areas with limited access to integrated care. The MIRACLE-AF trial demonstrated significant reductions in cardiovascular mortality, stroke, and heart failure or acute coronary syndrome hospitalizations with telemedicine-based, village doctor-led integrated management versus usual care. We conducted an economic evaluation of this management among patients aged ≥65 years in rural China. A Markov model was developed from the healthcare provider perspective to simulate a hypothetical cohort over a 15-year horizon. Outcomes included incremental cost-effectiveness ratios (ICERs) per cardiovascular death avoided and per stroke avoided, and incremental cost-utility ratios (ICURs) per quality-adjusted life-year (QALY) gained. Costs were expressed in 2024 US dollars ($1 = 7.1975 CNY), with a willingness-to-pay threshold of $35,718 (three times rural China’s per-capita GDP in 2024). Compared with usual care, telemedicine-based integrated yielded ICERs of $60,692 per cardiovascular mortality and $4,880 per stroke avoided, and a favorable ICUR of $4,554 per QALY gained. Probabilistic sensitivity analysis indicated cost-effective in 97.60% of simulations, with results most sensitive to the number of patients managed per village doctor. These findings support telemedicine-based, village doctor-led integrated AF management as a potential cost-effective strategy in resource-limited rural settings.

## Introduction

Atrial fibrillation (AF) is the most common arrhythmia, associated with increased risks of stroke, heart failure (HF), dementia, and mortality^1^. In China, AF has emerged as the fastest-growing cardiovascular disorder in terms of disease burden^2^, particularly among the elderly population. This trend is especially pronounced in rural areas, where the population is aging rapidly and the prevalence of AF continues to increase. However, awareness, diagnosis, and management of AF remain extremely inadequate in these settings^3^.

Compared to urban areas, rural China faces substantial systemic barriers to effective AF care, stemming from challenges on both the patients and healthcare providers. Elderly population in rural areas often have low incomes, limited educational attainment, and poor health literacy, restricting their access to health information and service^4–6^. Physical frailty and poor transportation infrastructure further isolate them from the broader healthcare system. On the provider side, village doctors—who deliver primary care for the majority of the rural population—are typically undertrained, underpaid, and aging themselves, with limited capacity to manage complex chronic conditions^7^. According to the China Health Statistical Yearbook 2022^8^, among the 671,298 village doctors in 2021, 74.9% were over 45 years old, and only 39.2% had received higher education. These multifactorial challenges make it difficult to implement guideline-recommended AF management especially difficult in rural communities, highlighting the urgent need for scalable interventions to strengthen rural healthcare system.

China’s rapid urbanization underscores the need for innovative health strategies including digital health technologies to reduce health inequities in rural areas^9^. However, older adults in rural areas often struggle to use digital platforms, and ensuring that they can benefit from these new medical opportunities and services in the long term rather than creating a new digital divide is of great significance to the assessment of health equity^10,11^.

To address these challenges, a telemedicine-based, village doctor-led integrated management^12^ was developed as a potential solution, incorporating AF specialist guidance and real-time digital support to enhance compliance with holistic, guideline-recommended care based on the Atrial fibrillation Better Care (ABC) pathway^13,14^. This intervention model focused on empowering village doctors through continuous remote support, enabling them to deliver integrated AF care aligned with the ABC pathway. By embedding digital tools into routine practice and linking village doctors with specialist teams, the model optimized the use of medical resources and improved care coordination.

Between December 1, 2020, and May 9, 2024, a cluster randomized controlled trial (MIRACLE-AF, ClinicalTrials.gov: NCT04622514)^15^ was conducted across 30 rural village clinics to evaluate the efficacy and safety of this telemedicine-based integrated management. The study enrolled 1039 patients with AF aged ≥65 years, managed by 31 village doctors, of whom 83.9% had completed vocational medical education and 93.5% held formal medical qualifications. Participants were randomized to receive either telemedicine-based, village doctor-led integrated management or usual care. Compared with usual care, the telemedicine-based integrated management significantly improved adherence to ABC pathway and improved clinical outcomes, specifically reducing the risk of cardiovascular death by 50%, while the rates of any stroke and hospitalization due to worsening of HF or acute coronary syndrome (ACS) were 36% and 31% lower, respectively. Hence, this telemedicine-based integrated management presents a promising strategy for transforming AF management in rural China^16^.

Large-scale implementation of this telemedicine-based integrated management would require extensive evaluation and substantial investment. A health economic assessment would be crucial to determine its economic feasibility, scalability, and long-term sustainability in resource-limited settings^17,18^. To address this, we constructed a Markov model to evaluate the potential cost-effectiveness and cost-utility of implementing this telemedicine-based integrated management for AF patients aged 65 and older in rural China, compared with usual care, from the health care provider’s perspective. The analysis was reported according to the Consolidated Health Economic Evaluation Reporting Standards 2022 (CHEERS 2022) statement (Supplementary Table 1)^19^.

## Results

### Base-case analysis

Under the base-case conditions, the cost-effectiveness and cost-utility analysis showed that implementation of telemedicine-based integrated AF management was highly cost-effective in rural China.

The estimated medical cost per 1000 patients was $1,831,713.16 under the telemedicine-based integrated management, compared to $1,514,514.13 under usual care. Implementation of the novel management is projected to reduce cardiovascular mortality by 51.16%, reduce stroke incidence by 32.10% (Table 1). The incremental cost-effectiveness ratios (ICERs) were $60,691.73 per cardiovascular mortality avoided and $4,880.20 per stroke avoided. The incremental cost-utility ratio (ICUR) was $4,554.18 per quality-adjusted life-year (QALY) gained, which was below the threshold of 3-time per-capita gross domestic product (GDP) ($35,718).

**Table 1:** Base-case cost-effectiveness and cost-utility results of different management strategies over a 15-year time horizon

Outcome	Usual care	Telemedicine-based management	Increment ^a^	Relative reduction ^b^
Cost				
Costs per 1000 patients	$1,514,514.13	$1,831,713.16	$317,199.03	−20.94%
Mean costs per patient	$1,514.51	$1,831.71	317.20	−20.94%
Effectiveness / utility				
Stroke cases per 1000 patients	202.49	137.50	−65.00	32.10%
Cardiovascular deaths per 1000 patients	10.21	4.99	−5.23	51.16%
QALYs per 1000 patients	7,062.25	7,131.90	69.65	−0.99%
Cost-effectiveness / cost-utility				
Incremental costs per stroke avoided^c^	reference	$4,880.20	–	–
Incremental costs per cardiovascular death avoided^d^	reference	$60,691.73	–	–
Incremental costs per QALY gained^e^	reference	$4,554.18	–	–

^a^The increment was calculated as outcome(telemedicine-based management)—outcome(usual care), using original unrounded values.

^b^The relative reduction was calculated as [outcome(usual care)—outcome(telemedicine-based management)] / outcome(usual care), using original unrounded values.

^c^The incremental costs per stroke avoided were calculated by dividing the incremental costs per 1000 patients by the number of strokes avoided per 1000 patients, using original unrounded values.

^d^The incremental costs per cardiovascular death avoided were calculated by dividing the incremental costs per 1000 patients by the number of cardiovascular deaths avoided per 1000 patients, using original unrounded values.

^e^The incremental costs per QALY gained were calculated by dividing the incremental costs per 1000 patients by the QALY gained per 1000 patients, using original unrounded values.

QALY quality-adjusted life-year.

### Sensitivity analysis

**Fig. 1:**
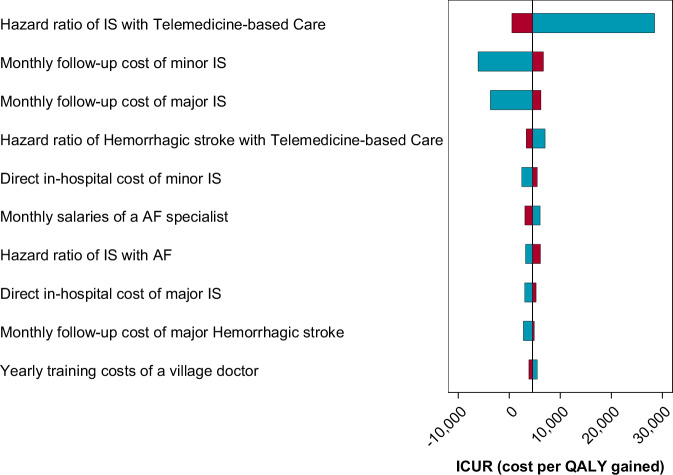
Deterministic one-way sensitivity analysis. We performed one-way sensitivity analyses for Telemedicine-based management versus usual care. The top 10 parameters with the greatest impact on ICURs are shown in above figure. Red indicates the lower parameter sensitivity input and blue indicates the higher parameter sensitivity input in Supplementary Table 3. Costs were expressed in 2024 US dollars ($1 = 7.1975 Chinese yuan). IS ischemic stroke, AF atrial fibrillation, ICUR incremental cost utility ratio, QALY quality-adjusted life-year.

Tornado diagrams from the one-way sensitivity analysis demonstrated the robustness of the base-case results across a wide range of input parameters. All ICURs remained below the WTP threshold of $35,718 per QALY gained (Fig. 1).

**Fig. 2:**
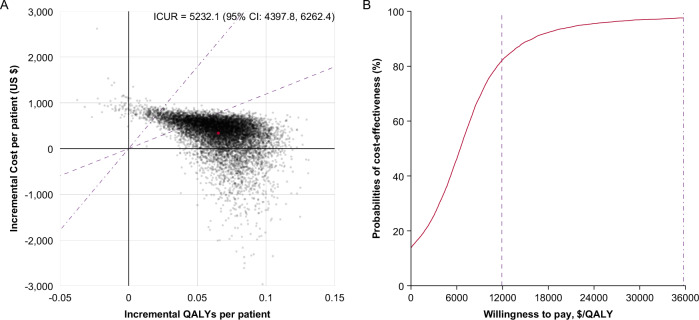
Cost-effectiveness plane and acceptability curve simulating all parameters. **A** Gray point in the cost-effectiveness plane represents one of 10,000 simulations of incremental cost and QALY pairs, varying all parameters in Supplementary Table 3. The red point indicates the base-case estimate. **B** The red acceptability curve shows the proportion of simulations deemed cost-effective across varying willingness-to-pay thresholds, indicating that telemedicine-based integrated management is cost-effective. The dashed and dot-dashed purple lines denote one-time and three-times rural China’s per-capita GDP in 2024 ($11,906 and $35,718), respectively. Costs were expressed in 2024 US dollars ($1 = 7.1975 Chinese yuan). QALY quality-adjusted life-year, GDP gross domestic product, ICUR incremental cost-utility ratio.

**Fig. 3:**
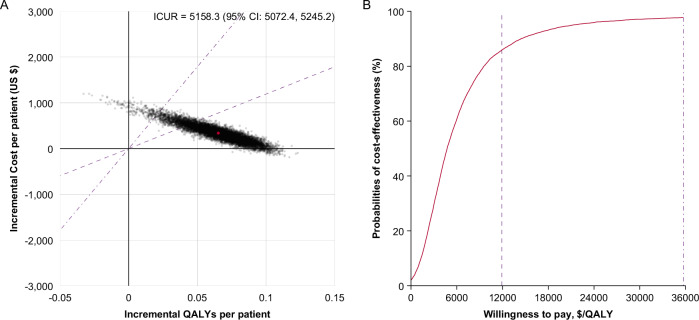
Cost-effectiveness plane and acceptability curve simulating strategy-related parameters (Scenario 1). **A** Gray point in the cost-effectiveness plane represents one of 10,000 simulations of incremental cost and QALY pairs, varying strategy-related parameters in Supplementary Table 3. The red point indicates the base-case estimate. **B** The red acceptability curve shows the proportion of simulations deemed cost-effective across varying willingness-to-pay thresholds, indicating that telemedicine-based integrated management is cost-effective. The dashed and dot-dashed purple lines denote one-time and three-times rural China’s per-capita GDP in 2024 ($11,906 and $35,718), respectively. Costs were expressed in 2024 US dollars ($1 = 7.1975 Chinese yuan). QALY quality-adjusted life-year, GDP gross domestic product, ICUR incremental cost-utility ratio.

Monto Carlo simulations based on all parameters (Fig. 2) and strategy-related model parameters (Fig. 3) demonstrated that telemedicine-based integrated management was cost-effective in 97.60% of simulations under the threshold of 3-time per-capita GDP ($35,718). The probabilistic analyses yielded an ICUR of $5,232.1 (95% CI: $4,397.8, $6,262.4, when varying all parameters) and $5,158.3 (95% CI: $5,072.4 to $5,245.2, when varying strategy-related parameters) per QALY gained. Varying discount rates from 0% to 8% yielded minimal changes ($4,827.0–$5,595.0 per QALY gained) (Supplementary Fig. 1-2). When considering healthcare delivery capacity in rural settings by reducing the number of patients managed by a village doctor, the ICURs became less favorable. Specifically, the ICUR increased to $9,196.0 per QALY gained for 25 AF patients per village doctor, $18,503.1 per QALY gained for 15 AF patients per village doctor and $30,105.8 per QALY gained for 10 AF patients per village doctor (Supplementary Fig. 3–5).

## Discussion

To the best of our knowledge, this is the first study to evaluate the economic sustainability of a telemedicine-based integrated AF management in rural China^16,20^. Our simulation suggests that, when compared with usual care, implementing a telemedicine-based, village doctor-led integrated management approach can reduce cardiovascular mortality and stroke incidence, improve QALYs, and save healthcare expenditures. In broad sensitivity analyses, the main findings remain robust to a wide range of changes in parameters.

Two major integrated care models for AF have been developed and evaluated in China: the mobile Health (mHealth) technology-supported mAFA II model^21^ targeting urban populations, and the telemedicine-based MIRACLE-AF model designed for rural settings. Previous health economic evaluations^22^ assessed the nationwide cost-effectiveness of mHealth–based integrated care using effectiveness data derived from mAFA II. However, the extrapolation of this effectiveness to rural areas remains uncertain due to lower smartphone penetration and digital literacy. The study reported an approximately 50% probability of cost-effectiveness at a willingness-to-pay threshold of one-time per-capita GDP. In contrast, using rural-specific clinical evidence from the MIRACLE-AF trial, our analysis shows that the telemedicine-based integrated management has an approximately 82.0% probability of being cost-effective at the similar threshold. These findings highlight the importance of context-specific evidence in economic evaluations.

The primary effectiveness assumption in our analysis is based on the reported findings from the MIRACLE-AF trial^15^, the first trial to evaluate a telemedicine-based integrated management for older AF patients in rural villages. In low- and middle-income countries, this vulnerable population often faces systemic healthcare disparities^5^, which limits the applicability of previously developed smartphone application-based integrated care models primarily designed for more educated and health-literate populations^21,23^. By providing more accessible basic medical training and leveraging telemedicine technology, the MIRACLE-AF model empowers village doctors to play a leading role in AF management. This approach helps bridge the gap between rural patients and AF specialists and contributes to long-term improvements in the rural primary healthcare system.

To strengthen primary healthcare, China officially launched the Family Doctor Contract Service (FDCS) in June 2016^24^. Under this policy, village doctors are responsible for providing contracted residents in rural areas with basic medical care and public health services, including chronic disease management^25^. Studies have confirmed the FDCS’s positive impact on healthcare access^26–28^, yet challenges remain in perceived service quality. A recent survey indicated that contracted residents expressed the lowest satisfaction with the professional skills of their family doctors^29^, highlighting the ongoing need to enhance the capabilities of primary care providers. To improve the quality of primary care and promote a more equitable distribution of health resources, China has introduced supporting policies aimed at developing medical consortiums and telemedicine collaboration networks for underserved and remote areas^30,31^. The telemedicine collaboration network has covered all cities and counties, and is extending to community and rural clinics. The findings of our health economics evaluation further support the practical value of these policies in promoting telemedicine-based care models.

This study has several strengths. First, we employed a Markov modeling framework to simulate the primary treatment goals of a telemedicine-based, village doctor-led integrated AF management model and to assess its two-sided effects. On the benefit side, by improving adherence to the guideline-recommended ABC pathway^13^, the intervention reduced the incidence of stroke, hospitalizations, and other adverse cardiovascular outcomes. On the potential harm side, one component of the ABC pathway—optimization of anticoagulation therapy—while essential for stroke prevention, may increase the risk of bleeding events^32,33^. Given the uncertainty surrounding real-world implementation across heterogeneous clinical settings, we incorporated an increased risk of ECH observed in the MIRACLE-AF trial (HR, 1.54; 95% CI, 0.882–2.691) to reflect the inherent trade-off between enhanced stroke prevention and bleeding complications. Second, the incidence data used in our study were derived from large-scale population studies representative of the general rural population, thereby enhancing the extrapolation and generalizability of our findings.

Nonetheless, our study has several limitations. First, our reliance on the MIRACLE-AF trial as the primary source of clinical probabilities may introduce bias, as the effectiveness estimates were derived from a relatively short follow-up period and extrapolated to lifetime horizon. The MIRACLE-AF trial was conducted in more affluent eastern regions of China, raising concerns about its generalizability to the central and western regions, where medical resources are more limited^34^. In these regions, factors such as physician engagement, the coverage of family doctor contracts, and the availability of healthcare infrastructure may influence the cost-effectiveness of the intervention model, as reflected in the scenario analyses presented in Supplementary Figs. 3–5^35^. Additionally, although the MIRACLE-AF trial conducted prespecified subgroup analyses—including age, sex, new or recent AF, education level, and CHA₂DS₂-VASc score—no statistically significant heterogeneity in treatment effects was observed across these subgroups. Due to the lack of comprehensive public surveillance data in rural China, we were unable to perform region-specific subgroup analyses. Second, the largest cost component in the model was stroke follow-up costs, which were based on follow-up data for first-ever stroke cases 12 months after discharge^36^. These follow-up costs during this period are relatively high^37^ and may lead to overestimation of long-term expenditures once the disease stabilizes. Third, pooling data from non-Chinese studies may introduce bias in demographics and baseline health status between global and rural Chinese populations. For example, the effects of different health states on utility were derived from multiple studies worldwide. However, given the scarcity of high-quality, rural-specific data in China, such approximations are often necessary in economic modeling. Fourth, we did not perform external validation^38^. Although our model demonstrated robust internal validity and logical consistency checks, the long-term outcomes predicted by the Markov model (e.g., 15-year stroke incidence) could not compared with independent real-world longitudinal data from the Chinese population because such registries are currently unavailable.

The Chinese government has been encouraging village doctors to provide long-term care for elderly rural patients with chronic diseases^39^. Building on clinical evidence, we supplemented the health economics evaluation of a telemedicine-based, village doctor-led integrated AF management that leveraged the established telemedicine collaboration network, thereby providing a theoretical foundation for policy promotion and implementation. Future economic evaluations conducted alongside randomized clinical trials are warranted to verify these findings and to explore key influencing factors, including geographical variation in patient characteristics, potential treatment-effect heterogeneity across subgroups, and constraints related to local implementation capacity.

## Methods

### Model overview

We constructed a discrete-time Markov model with 1-month cycles using R (version 4.4.1) and simulated a hypothetical cohort of 1000 AF patients aged ≥65 years, excluding those with prior valve replacement surgery, moderate to severe rheumatic mitral stenosis, implantable cardiac defibrillators or cardiac resynchronization therapy devices, previous catheter ablation of AF, left atrial appendage occlusion, or contraindications to anticoagulant therapy. Since our study was based on publicly available data, Ethics Committee Approval was not required.

**Fig. 4:**
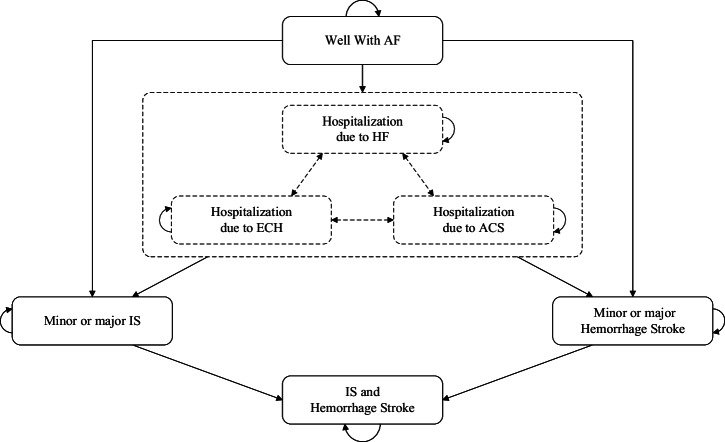
Markov model showing possible transitions between health states. Patients start out with uncomplicated AF, then cycle between health states until death occurs or the 15-year period ends. The length of each cycle is 30 days. The health states are equivalent for two management strategies, but the probabilities, costs, and quality of life vary with strategy. Any health state could lead to death (not shown). AF Atrial fibrillation, HF heart failure, ECH extracranial hemorrhage, ACS acute coronary syndrome, IS ischemic stroke.

The model structure was adapted from the previous studies evaluating anticoagulant treatment^40–44^ or management models^22^ in patients with AF (Fig. 4). It was further supplemented with related health states according to the goals of integrated AF management^45^. Permanent health states in the model included well with AF, minor and major ischemic stroke (IS), minor and major hemorrhage stroke, combined IS and hemorrhage stroke, and death. Temporary health states included hospitalization due to ACS, extracranial hemorrhage (ECH), and worsening of HF. Patients transitioned between health states based on the risk of clinical events, either moving from one state to another (e.g., an AF patient experiencing a stroke or death) or remaining in the same state (e.g., a patient staying well or remaining disabled after a stroke). The model was validated by simulating outcomes over a three-year period and comparing the predicted incidence of clinical outcomes with the corresponding data from the MIRACLE-AF trial (Supplementary Table 2)^15^.

Costs and utilities were applied to each of the outcomes over their expected duration. For each strategy, we projected risk of clinical events, net cost, and QALYs over 15 years. We applied an annual discount rate of 5% to costs and QALYs to reflect time preference but not to discrete clinical events^41,46^.

### Clinical parameters

All model inputs were listed (Supplementary Table 3), including a complete list of all parameter base-case inputs, ranges, distributions, and sources. Most transition data were obtained from published studies specific to rural China. The incidence of ACS was estimated based on trends in acute myocardial infarction incidence, as reported in a cross-sectional survey covering 307 consistent townships over a 12-year period^47^. Hospitalization rate for HF was estimated from a national population-based Study of an urban population^48^. Age-specific stroke incidence rates were calculated based on a nationwide cross-sectional study^49^, assuming that the age-specific and geographic (urban/rural) distribution patterns of stroke subtypes mirror those observed in the general population. The proportions of minor, major, and fatal strokes were estimated based on the modified Rankin Scale (mRS) score at discharge reported in the China stroke surveillance report 2021^50^. The stroke recurrence rates and recurrence subtype distributions were estimated from a prospective cohort study of 0.5 million Chinese adults^51^. Consistent with established model in stroke progression^42^, we assumed that a second minor stroke resulted in a major stroke and that a second major stroke resulted in death. Age-specific mortality were estimated from the 2020 Population Census of China^52^.

Previous studies have demonstrated the adverse effects of AF on cardiovascular events and all-cause mortality^1^. The specific hazard ratios (HRs) for these outcomes were derived from a prospective longitudinal cohort study^53^, except for ECH. By combining the event rates in the general rural population with the hazard ratios with AF, we estimated the event rates under usual AF care. Due to the lack of other published data on the incidence of ECH among Chinese patients with AF, we used the observed rate in the control group of the MIRACLE-AF trial as an estimate^15^. Stroke patients have a higher mortality rate than the general population, with the relative risk derived from the 20-year follow-up of the Framingham Study Cohort^54^. The effectiveness of telemedicine-based care versus usual care was retrieved from the MIRACLE-AF trial^15^, with HRs of 0.50 (95% CI: 0.32–0.80) for cardiovascular death, 0.642 (95% CI: 0.412–0.999) for stroke, 0.685 (95% CI: 0.493–0.953) for HF or ACS. To account for the potential adverse effects of optimized anticoagulation strategies on bleeding risk^32,33^, we incorporated an increased hazard for ECH, with a pooled HR of 1.54 (95% CI: 0.882–2.691), also derived from the MIRACLE-AF trial.

The incidence rates were converted into the monthly transition probabilities using the formula $p=1-{e}^{-r/t}$, where *p* denoted the monthly probability, and *r* represented the incidence rate over *t* months^55^.

### Costs

As our analysis focused on the incremental cost-effectiveness and cost-utility of the novel management compared with the current approach, we excluded costs unrelated to antithrombotic therapy, temporary in-hospital events, or stroke outcomes. All costs were expressed in 2024 US dollars^56^ ($1 = 7.1975 Chinese yuan).

The construction and monthly operation costs of the telemedicine platform were based on actual data and experts’ opinions from the MIRACLE-AF study, as were the renovation costs for the village clinics. For secondary or tertiary hospitals that provide telemedicine support, we also considered their renovation costs. The relevant data comes from a survey on the current status of telemedicine construction in hospitals in different regions of China^57^. We estimated the costs of the novel management strategy based on the data from a previous trial on chronic disease management, which was also led by village doctors^58^. The cost components included salaries and training expenses for village doctors and AF specialists. We assumed that each village doctor manages 35 AF patients and receives offline standardized training once a year and online training every month.

For AF patients without complications, routine antithrombotic therapy was the only cost component considered. Monthly cost estimates incorporated both the proportion of antithrombotic therapies in the rural population and the associated costs. The proportions under different management strategies were derived from the MIRACLE-AF study. Treatment costs were based on expert opinion regarding typical prescription patterns, current drug prices, and necessary monthly international normalized ratio (INR) testing for warfarin users.

Considering that the majority of related ECHs are gastrointestinal bleeding events^59^, we used the direct economic burden of acute upper gastrointestinal bleeding in rural areas to estimate the in-hospital cost of ECH^60^. The in-hospital costs of HF and ACS were estimated using the same data source.

The in-hospital and follow-up costs of IS came from the first real-world study investigating the follow-up costs of first-ever IS in China^36^, which provided detailed cost information for rural patients. The study also showed that, AF was not significantly associated with total costs. Therefore, we did not assign different costs for IS based on AF. Based on the national annual average in-hospital costs of hemorrhage stroke (¥18,543.4) and IS (¥6,960.3) in county-level hospitals^8^, we estimated the in-hospital costs of hemorrhage stroke. The follow-up costs of hemorrhage stroke were assumed to be the same as those for IS.

### Utilities

All utility values were derived from published studies based on EuroQol (EQ-5D) assessments. Death from any cause was assigned a utility value of 0. Disutilities associated with various clinical events were combined additively to estimate their overall impact on patients’ quality of life^41,61^.

The utility value for AF was obtained from a prospective, multicenter, nationwide registry study of elderly Chinese patients with AF^62^. Previous studies have demonstrated that ACS and ECH are associated with a temporary loss in health utility at the time of onset, without evidence of long-term impact^63,64^. Therefore, only temporary disutilities were assigned to these events and lasted only one month. Temporary disutility associated with hospitalization for worsening of HF was derived from a health economic analysis about the treatment of HF^65^. Permanent disutilities for stroke were obtained from a large international database^66^, which had been previously used in a multicenter clinical trial in China^67^. For IS, the utility values corresponding to 3-month mRS scores of 0 to 5 were 0.97, 0.89, 0.74, 0.57, 0.28, and −0.09, respectively. For hemorrhagic stroke—assuming that intracerebral and subarachnoid hemorrhage have the same effects—the corresponding values were 0.97, 0.88, 0.74, 0.55, 0.20, and −0.19, respectively. Based on the distribution of 3-month mRS scores in China^50^, we estimated the average disutilities for minor (mRS≤1) and major (mRS≥2) cases of each stroke subtype.

### Primary outcomes

The primary outcomes were the ICERs and ICUR, calculated using the following formulas:

1 $$\begin{array}{ll}ICE{R}_{Stroke} & =\frac{Costs\,per\,1000\,patients(telemedicine-based)-Costs\,per\,1000\,patients(usual)}{Stroke\,cases\,per\,1000\,patients(usual)-Stroke\,cases\,per\,1000\,patients(telemedicine-based)}\\ & =\frac{Incremental\,costs}{Stroke\,cases\,avoided}\end{array}$$

2 $$\begin{array}{ll}ICE{R}_{Death} & =\frac{Costs\,per\,1000\,patients(telemedicine-based)-Costs\,per\,1000\,patients(usual)}{Cardiovascular\,deaths\,per\,1000\,patients(usual)-Cardiovascular\,deaths\,per\,1000\,patients(telemedicine-based)}\\ & =\frac{Incremental\,costs}{Cardiovascular\,deaths\,avoided}\end{array}$$

3 $$\begin{array}{ll}ICUR & =\frac{Costs\,per\,1000\,patients(telemedicine-based)-Costs\,per\,1000\,patients(usual)}{QALYs\,per\,1000\,patients(telemedicine-based)-QALYs\,per\,1000\,patients(usual)}\\ & =\frac{Incremental\,costs}{QALYs\,gained}\end{array}$$

According to WHO guidelines^19^, interventions costing less than the per capita GDP are considered highly cost-effective; those costing less than three times the per capita GDP are deemed cost-effective. We estimated the 2024 per capita GDP for rural China to be $11,906, based on the national per capita GDP ($13,123), urbanization rate (0.67), and the urban-rural income ratio (2.34)^68,69^.

### Base-case analysis and sensitivity analysis

The base-case analysis was conducted using the most plausible central estimates for all model input parameters, representing the primary reference scenario against which alternative assumptions were compared^70,71^. It reflected neither an optimistic nor a pessimistic set of assumptions, but rather the most likely conditions based on the available evidence. Detailed parameter values used in the base-case analysis were provided in Supplementary Table 3. Results were presented as the ICERs and ICUR.

We performed extensive sensitivity analysis to assess the robustness of the primary results. One-way deterministic sensitivity analyses were conducted on each parameter using the bounds of its 95% CI to evaluate the stability of the results under key assumptions (Supplementary Table 3). Tornado diagrams showed the top ten parameters with the greatest influence on the ICURs.

Additionally, probabilistic sensitivity analyses (PSA) were performed using a Monte Carlo simulation with 10,000 sets of simulated parameters to account for joint uncertainty in costs and QALYs. Unless otherwise specified, uncertainty was assigned to all model input parameters listed in Supplementary Table 3, including clinical probabilities, costs, and health utility values. The discount rate was fixed at 5%, and each village doctor was assumed to manage 35 patients with AF. Beta distributions were assigned to transition probabilities, gamma distributions to costs and utility parameters, and log-normal distributions to intervention effectiveness hazard ratios^72^. PSA results were presented as cost-effectiveness acceptability curves and the 95% central ranges for health benefits were estimated from these simulations^73^.

Several PSA scenarios were considered. In Scenario 1, uncertainty was restricted to parameters directly related to the telemedicine-based integrated management^74^, as marked in Supplementary Table 3, including strategy-specific costs, implementation-related resource use, and effectiveness estimates, while all other parameters were held constant at their base-case values. In Scenario 2 and Scenario 3, alternative annual discount rates of 0% and 8%, respectively, were applied to both costs and health outcomes, in accordance with health economic evaluation guidelines in China^46^. Finally, to examine uncertainty related to healthcare delivery capacity in rural settings, additional scenarios were evaluated assuming 25 (Scenario 4), 15 (Scenario 5) and 10 (Scenario 6) AF patients per village doctor. The values of 15 and 10 patients correspond to the lower bounds of the 95% and 99% CIs, respectively, derived from empirical observations in the MIRACLE-AF trial^15^.

## Supplementary information

Supplementary Information

## Data Availability

All data used to construct the model is publicly available and referenced. All parameters and their values can be found in Supplementary Tables 3 and Supplementary References. Any additional data is available upon request from the corresponding author.

## Code availability

The code that supports the findings of this study is available from the corresponding author upon reasonable request.
