# Supplementary material for: Economic evaluation of telemedicine-based integrated management of atrial fibrillation in rural China: a modeling analysis
Source: NPJ Digit Med. 2026 May 19;9:612. doi: 10.1038/s41746-026-02772-6 (PMC13454166; doi:10.1038/s41746-026-02772-6)
Supplement: Supplementary file 1 [file 41746_2026_2772_MOESM1_ESM.pdf]

Supplementary Table 1. The CHEERS 2022 checklist.

Section	Item No	Recommendation	Reported on page No/line No
**Title**			
Title	1	Identify the study as an economic evaluation and specify the interventions being compared.	1
**Abstract**			
Abstract	2	Provide a structured summary that highlights context, key methods, results, and alternative analyses.	2
**Introduction**			
Background and objectives	3	Give the context for the study, the study question, and its practical relevance for decision making in policy or practice	3-4
**Methods**			
Health economic analysis plan	4	Indicate whether a health economic analysis plan was developed and where available.	No separate pre-specified health economic analysis plan was developed. Relevant methods are described in the Methods section (9, 13-14)
Study population	5	Describe characteristics of the study population (such as age range, demographics, socioeconomic, or clinical characteristics).	9
Setting and location	6	Provide relevant contextual information that may influence findings.	10-13
Comparators	7	Describe the interventions or strategies being compared and why chosen.	4
Perspective	8	State the perspective(s) adopted by the study and why chosen.	4
Time horizon	9	State the time horizon for the study and why appropriate.	9
Discount rate	10	Report the discount rate(s) and reason chosen.	9
Selection of outcomes	11	Describe what outcomes were used as the measure(s) of benefit(s) and harm(s).	13
Measurement of outcomes	12	Describe how outcomes used to capture benefit(s) and harm(s) were measured.	13
Valuation of outcomes	13	Describe the population and methods used to measure and value outcomes.	9
Measurement and valuation of resources and costs	14	Describe how costs were valued.	11-12, Supplementary Table 3
Currency, price date, and conversion	15	Report the dates of the estimated resource quantities and unit costs, plus the currency and year of conversion.	11
Rationale and description of model	16	If modelling is used, describe in detail and why used. Report if the model is publicly available and where it can be accessed.	9

Analytics and assumptions	17	Describe any methods for analyzing or statistically transforming data, any extrapolation methods, and approaches for validating any model used.	9
Characterising heterogeneity	18	Describe any methods used for estimating how the results of the study vary for subgroups.	NA
Characterising distributional effects	19	Describe how impacts are distributed across different individuals or adjustments made to reflect priority populations.	NA
Characterising uncertainty	20	Describe methods to characterise any sources of uncertainty in the analysis.	13-14, Supplementary Table 3
Approach to engagement with patients and others affected by the study	21	Describe any approaches to engage patients or service recipients, the general public, communities, or stakeholders (such as clinicians or payers) in the design of the study.	NA
**Results**			
Study parameters	22	Report all analytic inputs (such as values, ranges, references) including uncertainty or distributional assumptions.	10-13, Supplementary Table 3
Summary of main results	23	Report the mean values for the main categories of costs and outcomes of interest and summarise them in the most appropriate overall measure.	5
Effect of uncertainty	24	Describe how uncertainty about analytic judgments, inputs, or projections affect findings. Report the effect of choice of discount rate and time horizon, if applicable.	5-6 Figure 1-3 Supplementary Figure 1-5
Effect of engagement with patients and others affected by the study	25	Report on any difference patient/service recipient, general public, community, or stakeholder involvement made to the approach or findings of the study	NA
**Discussion**			
Study findings, limitations, generalisability, and current knowledge	26	Report key findings, limitations, ethical or equity considerations not captured, and how these could affect patients, policy, or practice.	6-9
**Other**			
Source of funding	27	Describe how the study was funded and any role of the funder in the identification, design, conduct, and reporting of the analysis	15
Conflicts of interest	28	Report authors conflicts of interest according to journal or International Committee of Medical Journal Editors requirements.	15

NA= Not applicable

From: Husereau D, Drummond M, Augustovski F, et al. Consolidated health economic evaluation reporting standards 2022 (CHEERS 2022) statement: updated reporting guidance for health economic evaluations. *BMJ*. 2022;376.

Supplementary Table 2. Model validation by comparing clinical outcomes from MIRACLE-AF trial data to model output.

Outcomes	Telemedicine-based Care			Usual Care		
	**Trial**	**Model**	**Relative difference (%)** [a]	**Trial**	**Model**	**Relative difference (%)**
Non-cardiovascular death	5.2%	5.35%	2.9	2.8%	2.64%	-5.7
Death caused by stroke	0.50%	0.495%	-1	0.87%	0.951%	9.3
All strokes	1.5%	1.53%	2	2.4%	2.32%	-3.3
Hospitalization due to worsening of HF or ACS	3.2%	3.06%	4.4	4.7%	4.31%	-8.3
ECH	1.68%	1.60%	-4.8	1.09%	1.04%	-4.6

The model was validated by simulating outcomes over a three-year period and comparing the predicted incidence of first stroke with the corresponding data from the MIRACLE-AF trial. The simulated three-year stroke event rates under the MIRACLE-AF strategy and usual care closely matched the observed outcomes in the MIRACLE-AF trial, with all relative deviations within 10%. HF heart failure. ECH extracranial hemorrhage. ACS acute coronary syndrome.

[a] Relative difference was defined as the difference between the model-output rate and the trial-data rate, divided by the trial-data rate.

Supplementary Table 3. Model variables: base-case values and 95% CIs used in sensitivity analyses.

Variables	Base case value (95% CI)	Distribution	References	Notes
**Clinical parameters**				
Prevalence of AF in rural China (⩾65 y)	0.056 (0.052, 0.061)	Beta (473.321, 7978.84)	(Zhang et al., 2025, https://doi.org/10.26599/1671-5411.2025.02.001)	P249
Incidence rate of ECH	0.0109 (0.0061, 0.0181)	Beta (11.181, 1014.58)	(Chu et al., 2025, https://doi.org/10.1038/s41591-025-03511-2)	Table 3<br>Major bleeding: 0.8% per year<br>Clinically relevant nonmajor bleeding 0.3% per year
Hospitalization rate of HF	0.0108 (0.0107, 0.0109)	Beta (44522.62, 4077942.09)	(H. Wang et al., 2021, https://doi.org/10.1161/CIRCHEARTFAILURE.121.008406)	Table 1, Table 3<br>48549/(2.1+1.3+1.1)*1000000
Incidence rate of ACS	0.0055 (0.0043, 0.0068)	Beta (75.932, 13729.90)	(Chang et al., 2021, https://doi.org/10.3390/ijerph182312276)	Table 1 Median IQR<br>Males: 645.2 (506.0, 831.5) per 100,000<br>Females: 445.9 (315.8, 574.5) per 100,000

Incidence rate of IS (65-69 y)	0.0051 (0.0048, 0.0054)	Beta (1141.29, 222028.66)	(Tu et al., 2023, https://doi.org/10.1001/jamanetworkopen.2023.1455)	Table 1<br>IR: 666.1 (629.8-702.4) per 100,000<br>Figure 3<br>Urban vs Rural: OR=1.24<br>IS Proportion: 77.7%
Incidence rate of IS (70-79 y)	0.0104 (0.0098, 0.0109)	Beta (1314.81, 125609.25)	(Tu et al., 2023, https://doi.org/10.1001/jamanetworkopen.2023.1455)	Table 1<br>IR: 1338.1 (1270.5-1405.7) per 100,000<br>Figure 3<br>Urban vs Rural: OR=1.24<br>IS Proportion: 77.7%
Incidence rate of IS (80+ y)	0.0213 (0.0200, 0.0227)	Beta (898.206, 41211.78)	(Tu et al., 2023, https://doi.org/10.1001/jamanetworkopen.2023.1455)	Table 1<br>IR: 2749.4 (2575.2-2923.7) per 100,000<br>Figure 3<br>Urban vs Rural: OR=1.24<br>IS Proportion: 77.7%

Incidence rate of Hemorrhagic stroke (65-69 y)	0.0008 (0.0007, 0.0008)	Beta (1147.30, 1473531.79)	(Tu et al., 2023, https://doi.org/10.1001/jamanetworkopen.2023.1455)	Table 1<br>IR: 666.1 (629.8-702.4) per 100,000<br>Figure 3<br>Urban vs Rural: OR=1.24<br>Stroke Proportion: 22.3%
Incidence rate of Hemorrhagic stroke (70-79 y)	0.0016 (0.0015, 0.0017)	Beta (1317.34, 835088.27)	(Tu et al., 2023, https://doi.org/10.1001/jamanetworkopen.2023.1455)	Table 1<br>IR: 1338.1 (1270.5-1405.7) per 100,000<br>Figure 3<br>Urban vs Rural: OR=1.24<br>Stroke Proportion: 22.3%
Incidence rate of Hemorrhagic stroke (80+ y)	0.0032 (0.0030, 0.0035)	Beta (923.416, 283730.23)	(Tu et al., 2023, https://doi.org/10.1001/jamanetworkopen.2023.1455)	Table 1<br>IR: 2749.4 (2575.2-2923.7) per 100,000<br>Figure 3<br>Urban vs Rural: OR=1.24<br>Stroke Proportion: 22.3%

Proportion of minor IS	0.62	NA	(Tu & Wang, 2023, https://doi.org/10.1186/s40779-023-00463-x)	Fig. S10
Proportion of major IS	0.373	NA	(Tu & Wang, 2023, https://doi.org/10.1186/s40779-023-00463-x)	Fig. S10
Proportion of fatal IS	0.007	NA	(Tu & Wang, 2023, https://doi.org/10.1186/s40779-023-00463-x)	Fig. S10
Proportion of minor Hemorrhagic stroke	0.334	NA	(Tu & Wang, 2023, https://doi.org/10.1186/s40779-023-00463-x)	Fig. S11, Fig. S12
Proportion of major Hemorrhagic stroke	0.62	NA	(Tu & Wang, 2023, https://doi.org/10.1186/s40779-023-00463-x)	Fig. S11, Fig. S12
Proportion of fatal Hemorrhagic stroke	0.046	NA	(Tu & Wang, 2023, https://doi.org/10.1186/s40779-023-00463-x)	Fig. S11, Fig. S12
5-year cumulative recurrent rate of IS	0.39 (0.38, 0.4)	Beta (3579.81, 5599.19)	(Chen et al., 2020, https://doi.org/10.1016/S2214-109X(20)30069-3)	Web Figure 2
5-year cumulative recurrent rate of Hemorrhagic stroke	0.39 (0.37, 0.42)	Beta (400.855, 626.978)	(Chen et al., 2020, https://doi.org/10.1016/S2214-109X(20)30069-3)	Web Figure 2

Proportion of recurrent IS after a first IS	0.86 (0.8517, 0.8681)	Beta (5868.98, 955.416)	(Chen et al., 2020, https://doi.org/10.1016/S2214-109X(20)30069-3)	Web Table 7
Proportion of recurrent IS after a first Hemorrhagic stroke	0.355 (0.3359, 0.3743)	Beta (846.023, 1537.14)	(Chen et al., 2020, https://doi.org/10.1016/S2214-109X(20)30069-3)	Web Table 7
Mortality rate (65-69 y)	0.0138 (0.0137, 0.0138)	Beta (467988.66, 33534660.57)	(National Bureau of Statistics of China, 2021)	Table 6-4c
Mortality rate (70-74 y)	0.0230 (0.0230, 0.0231)	Beta (537876.03, 22831273.47)	(National Bureau of Statistics of China, 2021)	Table 6-4c
Mortality rate (75-79 y)	0.0400 (0.0399, 0.0401)	Beta (597512.95, 14334785.77)	(National Bureau of Statistics of China, 2021)	Table 6-4c
Mortality rate (80-84 y)	0.0704 (0.0702, 0.0705)	Beta (657953.77, 8692655.77)	(National Bureau of Statistics of China, 2021)	Table 6-4c
Mortality rate (85-89 y)	0.1120 (0.1117, 0.1122)	Beta (547060.78, 4339395.57)	(National Bureau of Statistics of China, 2021)	Table 6-4c
RR of mortality with stroke	1.14 (0.96, 1.35)	Log-Normal (0.129641, 0.086973)	(Gresham et al., 1998, https://doi.org/10.1161/01.STR.29.4.793)	Table 1<br>Stroke survivors mortality:92.5%<br>Age- and sex-matched controls mortality: 81%
HR of HF with AF	4.17 (3.45, 5.04)	Log-Normal (1.428, 0.096694)	(Yang et al., 2024, https://doi.org/10.1186/s12889-024-20703-6)	Table 4

HR of ACS with AF	1 (0.62, 1.62)	Log-Normal (0.002195, 0.245020)	(Yang et al., 2024, https://doi.org/10.1186/s12889-024-20703-6)	Table 4
HR of IS with AF	1.55 (1.24, 1.92)	Log-Normal (0.433718, 0.111536)	(Yang et al., 2024, https://doi.org/10.1186/s12889-024-20703-6)	Table 4
HR of Hemorrhagic stroke with AF	1.4 (0.84, 2.33)	Log-Normal (0.335757, 0.260265)	(Yang et al., 2024, https://doi.org/10.1186/s12889-024-20703-6)	Table 4
HR of all-cause mortality with AF	1.77 (1.61, 1.95)	Log-Normal (0.572032, 0.048877)	(Yang et al., 2024, https://doi.org/10.1186/s12889-024-20703-6)	Table 4
HR of ECH with Telemedicine-based AF Care [b]	1.54 (0.882, 2.691)	Log-Normal (0.432175, 0.284565)	(Chu et al., 2025, https://doi.org/10.1038/s41591-025-03511-2)	Table 3<br>Major bleeding: 1.17 (0.65, 2.13)<br>Clinically relevant nonmajor bleeding 2.94 (0.86, 10.03)
HR of HF with Telemedicine-based Care [b]	0.685 (0.493, 0.953)	Log-Normal (-0.377693, 0.168142)	(Chu et al., 2025, https://doi.org/10.1038/s41591-025-03511-2)	Table 3
HR of ACS with Telemedicine-based Care [b]	0.685 (0.493, 0.953)	Log-Normal (-0.377693, 0.168142)	(Chu et al., 2025, https://doi.org/10.1038/s41591-025-03511-2)	Table 3

HR of IS with Telemedicine-based Care [b]	0.642 (0.412, 0.999)	Log-Normal (-0.443866, 0.225956)	(Chu et al., 2025, https://doi.org/10.1038/s41591-025-03511-2)	Table 3
HR of Hemorrhagic stroke with Telemedicine-based Care [b]	0.642 (0.412, 0.999)	Log-Normal (-0.443866, 0.225956)	(Chu et al., 2025, https://doi.org/10.1038/s41591-025-03511-2)	Table 3
HR of Cardiovascular death with Telemedicine-based Care [b]	0.50 (0.32, 0.80)	Log-Normal (-0.681289, 0.233752)	(Chu et al., 2025, https://doi.org/10.1038/s41591-025-03511-2)	Table 3
**Cost parameters**[a]				
Platform construction costs [b]	27787.4262 (20840.5696, 34734.2827)	Normal (27787.43, 3544.38)	Experts' opinion	NA
Monthly platform operating expenses [b]	555.7485 (486.2800, 625.2171)	Normal (555.749, 35.444)	Experts' opinion	NA
Renovation costs for village clinics receiving telemedicine support, per village doctor [b]	2084.0570 (1389.3713, 2778.7426)	Normal (2084.06, 354.438)	Experts' opinion	NA
Renovation costs for the hospital providing telemedicine support, per hospital [b]	13893.7131 (7840.4485, 21650.1778)	Gamma (15.379, 903.439)	(Ye et al., 2025)	Table 1
Yearly training costs of a village doctor [b]	301.4489 (156.9960, 492.2354)	Gamma (12.250, 24.608)	(He et al., 2023, https://doi.org/10.1016/S0140-6736(22)02603-4)	Table S9
Monthly training costs of a village doctor [b]	25.1207 (13.0830, 41.0196)	Gamma (12.250, 2.051)	(He et al., 2023, https://doi.org/10.1016/S0140-6736(22)02603-4)	Table S9
Monthly salaries of a village doctor, per patient [b]	1.1587 (0.6562, 1.8017)	Gamma (15.548, 0.074522)	(He et al., 2023, https://doi.org/10.1016/S0140-6736(22)02603-4)	Table S9
Monthly salaries of an AF specialist [b]	135.1032 (101.3274, 168.8790)	Normal (135.103, 17.233)	Experts' opinion	NA

Monthly cost of Warfarin and INR testing	3.5846 (3.0844, 4.0848)	Normal (3.585, 0.255195)	Experts' opinion	NA
Monthly cost of Rivaroxaban	1.4727 (1.2754, 1.6686)	Normal (1.473, 0.100306)	Experts' opinion	NA
Monthly cost of Aspirin	2.0841 (1.8062, 2.3619)	Normal (2.084, 0.141775)	Experts' opinion	NA
Proportion of using Warfarin (Intervention) [b]	0.0038 (0, 0.0091)	Beta (3.032, 794.800)	(Chu et al., 2025, https://doi.org/10.1038/s41591-025-03511-2)	Table 2 Proportion of patients who met the A criterion
Proportion of using Rivaroxaban (Intervention) [b]	0.683 (0.6462, 0.7198)	Beta (405.420, 188.167)	(Chu et al., 2025, https://doi.org/10.1038/s41591-025-03511-2)	Table 2 Proportion of patients who met the A criterion
Proportion of using Aspirin (Intervention) [b]	0.0076 (0.0015, 0.0137)	Beta (7.921, 1034.35)	(Chu et al., 2025, https://doi.org/10.1038/s41591-025-03511-2)	Table 2 Proportion of patients who met the A criterion
Proportion of using Warfarin (Usual care)	0.0914 (0.0747, 0.1108)	Beta (84.369, 838.706)	(Chu et al., 2025, https://doi.org/10.1038/s41591-025-03511-2)	Table 2 Proportion of patients who met the A criterion
Proportion of using Rivaroxaban (Usual care)	0.0116 (0.0065, 0.0203)	Beta (8.892, 757.647)	(Chu et al., 2025, https://doi.org/10.1038/s41591-025-03511-2)	Table 2 Proportion of patients who met the A criterion
Proportion of using Aspirin (Usual care)	0.2205 (0.196, 0.2466)	Beta (222.208, 785.539)	(Chu et al., 2025, https://doi.org/10.1038/s41591-025-03511-2)	Table 2

				Proportion of patients who met the A criterion
Direct in-hospital cost of ECH	1031.8 (296.2478, 2217.9175)	Gamma (4.258, 242.295)	(Song et al., 2021, https://doi.org/10.3389/fpubh.2021.649624)	Table 2
Direct in-hospital cost of HF	1005.15 (276.2837, 2196.0245)	Gamma (4.041, 248.719)	(Song et al., 2021, https://doi.org/10.3389/fpubh.2021.649624)	Table 2
Direct in-hospital cost of ACS	2133.82 (1195.6532, 3339.2186)	Gamma (15.052, 141.765)	(Song et al., 2021, https://doi.org/10.3389/fpubh.2021.649624)	Table 2
Direct in-hospital cost of minor IS	2956.5238 (245.6487, 8952.8524)	Gamma (1.612, 1834.32)	(Lv et al., 2024, https://doi.org/10.1136/svn-2023-002296)	Supplement Table 2 Rural cooperation medical insurance Table 4 3-month mRS score 3–5 points OR=0.81
Monthly follow-up cost of minor IS	109.4792 (0.0043, 656.4997)	Gamma (0.338864, 323.077)	(Lv et al., 2024, https://doi.org/10.1136/svn-2023-002296)	Supplement Table 2 Rural cooperation medical insurance Table 4 3-month mRS score

				3–5 points<br>OR=0.81
Direct in-hospital cost of major IS	3649.9569 (303.1747, 11053.3501)	Gamma (1.612, 2264.89)	(Lv et al., 2024, https://doi.org/10.1136/svn-2023-002296)	Supplement Table 2<br>Rural cooperation medical insurance<br>Table 4<br>3-month mRS score<br>3–5 points<br>OR=0.81
Monthly follow-up cost of major IS	135.0895 (0.0053, 810.0495)	Gamma (0.338887, 398.627)	(Lv et al., 2024, https://doi.org/10.1136/svn-2023-002296)	Supplement Table 2<br>Rural cooperation medical insurance<br>Table 4<br>3-month mRS score<br>3–5 points<br>OR=0.81
Direct in-hospital cost of minor Hemorrhagic stroke	7876.6344 (654.3982, 23852.1326)	Gamma (1.612, 4887.09)	(Lv et al., 2024, https://doi.org/10.1136/svn-2023-002296; National Health Commission, 2022)	Reference 1<br>Supplement Table 2<br>Rural cooperation medical insurance<br>Table 4<br>3-month mRS score

				3–5 points<br>OR=0.81<br><br>Reference 2<br>Table 4-6-2
Monthly follow-up cost of minor Hemorrhagic stroke	109.4792 (0.0043, 656.4997)	Gamma (0.338864, 323.077)	(Lv et al., 2024, https://doi.org/10.1136/svn-2023-002296; National Health Commission, 2022)	Reference 1<br>Supplement Table 2<br>Rural cooperation medical insurance<br>Table 4<br>3-month mRS score<br>3–5 points<br>OR=0.81<br><br>Reference 2<br>Table 4-6-2
Direct in-hospital cost of major Hemorrhagic stroke	9724.0952 (807.7005, 29448.0279)	Gamma (1.612, 6034.08)	(Lv et al., 2024, https://doi.org/10.1136/svn-2023-002296; National Health Commission, 2022)	Reference 1<br>Supplement Table 2<br>Rural cooperation medical insurance<br>Table 4<br>3-month mRS score<br>3–5 points<br>OR=0.81

				Reference 2<br>Table 4-6-2
Monthly follow-up cost of major Hemorrhagic stroke	135.0895 (0.0053, 810.0495)	Gamma (0.338887, 398.627)	(Lv et al., 2024, https://doi.org/10.1136/svn-2023-002296; National Health Commission, 2022)	Reference 1<br>Supplement Table 2<br>Rural cooperation medical insurance<br>Table 4<br>3-month mRS score 3–5 points<br>OR=0.81<br><br>Reference 2<br>Table 4-6-2
**Utility parameters**				
Utility of Event-free AF [c]	0.827 (0.821, 0.832)	Gamma (861.303, 0.000948)	(Kotalczyk et al., 2022, https://doi.org/10.1161/JAHA.121.024319)	Table 1
Temporary disutility of ECH	0.029 (0.014, 0.044)	Gamma (0.995745, 0.013150)	(K. Wang et al., 2017, https://doi.org/10.1161/JAHA.117.006703)	P5
Temporary disutility of HF	0.1 (0.07, 0.13)	Gamma (41.567, 0.002370)	(King et al., 2016, https://doi.org/10.1016/j.jchf.2016.02.007)	P395

Temporary disutility of ACS	0.1535 (0.1035, 0.2035)	Gamma (35.093, 0.004298)	(Levin et al., 2013, https://doi.org/10.1016/j.jval.2013.01.013)	P573
Permanent disutility of minor IS	0.0701 (0.0691, 0.071)	Gamma (69.502, 0.000933)	(X. Wang et al., 2020, https://doi.org/10.1161/STROKEAHA.119.028523; Tu & Wang, 2023, https://doi.org/10.1186/s40779-023-00463-x)	Reference 1<br>Figure 2<br>3-month mRS score<br>0 points 0.97(0.10)<br>1 points 0.88 (0.16)<br>2 points 0.72(0.21)<br>3 points 0.54(0.25)<br>4 points 0.23(0.33)<br>5 points -0.17(0.21)<br><br>Reference 2<br>Table 4
Permanent disutility of major IS	0.4357 (0.4321, 0.4393)	Gamma (473.708, 0.000903)	(X. Wang et al., 2020, https://doi.org/10.1161/STROKEAHA.119.028523; Tu & Wang, 2023, https://doi.org/10.1186/s40779-023-00463-x)	Reference 1<br>Figure 2<br>3-month mRS score<br>0 points 0.97(0.10)<br>1 points 0.88 (0.16)<br>2 points 0.72(0.21)<br>3 points 0.54(0.25)<br>4 points 0.23(0.33)<br>5 points -0.17(0.21)<br><br>Reference 2

				Table 4
Permanent disutility of minor Hemorrhagic stroke	0.0713 (0.0686, 0.0739)	Gamma (77.731, 0.000859)	(X. Wang et al., 2020, https://doi.org/10.1161/STROKEAHA.119.028523; Tu & Wang, 2023, https://doi.org/10.1186/s40779-023-00463-x)	Reference 1<br>Figure 2<br>3-month mRS score<br>0 points 0.97(0.08)<br>1 points 0.88 (0.15)<br>2 points 0.74(0.16)<br>3 points 0.55(0.23)<br>4 points 0.20(0.31)<br>5 points -0.19(0.21)<br><br>Reference 2<br>Table 4
Permanent disutility of major Hemorrhagic stroke	0.5302 (0.5214, 0.539)	Gamma (535.276, 0.000973)	(X. Wang et al., 2020, https://doi.org/10.1161/STROKEAHA.119.028523; Tu & Wang, 2023, https://doi.org/10.1186/s40779-023-00463-x)	Reference 1<br>Figure 2<br>3-month mRS score<br>0 points 0.97(0.08)<br>1 points 0.88 (0.15)<br>2 points 0.74(0.16)<br>3 points 0.55(0.23)<br>4 points 0.20(0.31)<br>5 points -0.19(0.21)<br><br>Reference 2<br>Table 4

Utility of ECH	0.798 (0.782, 0.814)	Gamma(9587.40, 0.0000832)	(K. Wang et al., 2017, https://doi.org/10.1161/JAHA.117.006703)	Calculated [c]
Utility of HF	0.7271 (0.697, 0.7579)	Gamma(2182.636, 0.0003330835)	(King et al., 2016, https://doi.org/10.1016/j.jchf.2016.02.007)	Calculated [c]
Utility of ACS	0.6736 (0.6243, 0.7248)	Gamma(688.6902, 0.0009779433)	(Levin et al., 2013, https://doi.org/10.1016/j.jval.2013.01.013)	Calculated [c]
Utility of minor IS	0.7569 (0.7514, 0.7625)	Gamma(70647.41, 0.00001071377)	(X. Wang et al., 2020, https://doi.org/10.1161/STROKEAHA.119.028523; Tu & Wang, 2023, https://doi.org/10.1186/s40779-023-00463-x)	Calculated [c]
Utility of major IS	0.3913 (0.3848, 0.3979)	Gamma(13612.8, 0.000028745)	(X. Wang et al., 2020, https://doi.org/10.1161/STROKEAHA.119.028523; Tu & Wang, 2023, https://doi.org/10.1186/s40779-023-00463-x)	Calculated [c]
Utility of minor Hemorrhagic stroke	0.7557 (0.7496, 0.7618)	Gamma(58860.3, 0.00001283887)	(X. Wang et al., 2020, https://doi.org/10.1161/STROKEAHA.119.028523; Tu & Wang, 2023, https://doi.org/10.1186/s40779-023-00463-x)	Calculated [c]
Utility of major Hemorrhagic stroke	0.2968 (0.2866, 0.3073)	Gamma(3142.422, 0.00009444943)	(X. Wang et al., 2020, https://doi.org/10.1161/STROKEAHA.119.028523; Tu & Wang, 2023, https://doi.org/10.1186/s40779-023-00463-x)	Calculated [c]
Utility of minor IS plus major hemorrhagic stroke	0.2267 (0.2165, 0.2372)	Gamma(1823.969, 0.0001242894)	(X. Wang et al., 2020, https://doi.org/10.1161/STROKEAHA.119.028523; Tu & Wang, 2023, https://doi.org/10.1186/s40779-023-00463-x)	Calculated [c]

Utility of minor hemorrhagic stroke plus major IS	0.32 (0.313, 0.3271)	Gamma(7840.938, 0.00004081145)	(X. Wang et al., 2020, https://doi.org/10.1161/STROKEAHA.119.028523; Tu & Wang, 2023, https://doi.org/10.1186/s40779-023-00463-x)	Calculated [c]

[a] These variables were strategy-related and discussed in the probabilistic analysis.

[b] Costs are given in 2024 US dollars ($1 = 7.1975 Chinese yuan)

[c] The utility of the event-free AF health state was specified directly as an independent model input. Utilities of all other health states were treated as derived quantities and calculated by subtracting event-specific disutilities from the utility of the event-free AF state.

Abbreviations: AF=Atrial fibrillation. HF=heart failure. ECH=extracranial hemorrhage. ACS=acute coronary syndrome. IS=ischemic stroke. HR=hazard ratio. RR=relative risk. INR=international normalized ratio. NA=not applicable.

Supplementary Figure 1. Cost-effectiveness plane and acceptability curve simulating all parameters (Scenario 2: discount rate = 0%).

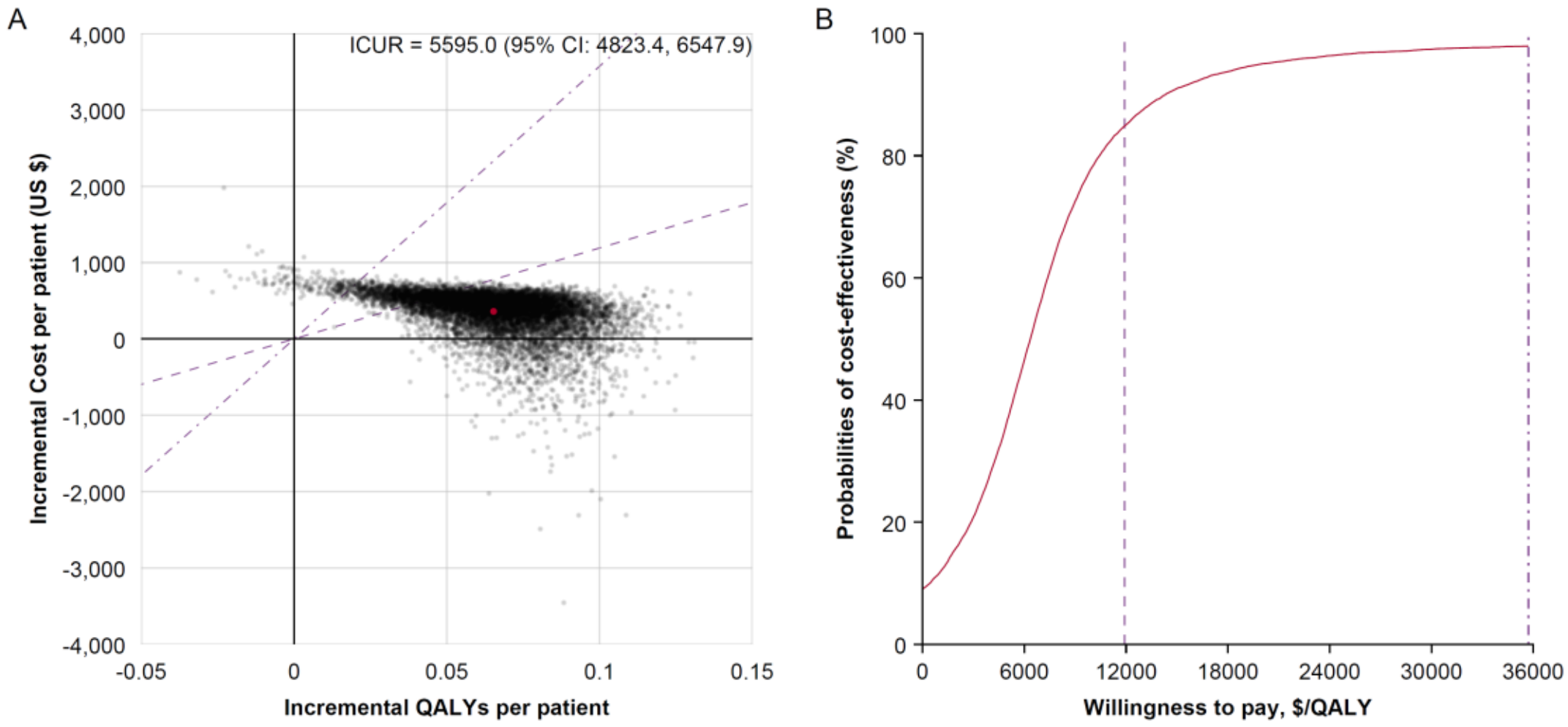

A Grey point in the cost-effectiveness plane represents one of 10,000 simulations of incremental cost and QALY pairs, varying all parameters in Supplementary Table 3. The red point indicates the base-case estimate. **B** The red acceptability curve shows the proportion of simulations deemed cost-effective across varying willingness-to-pay thresholds, indicating that telemedicine-based integrated management is cost-effective. The dashed and dot-dashed purple lines denote one-time and three-times rural China's per-capita GDP in 2024 ($11,906 and $35,718), respectively. Costs were expressed in 2024 US dollars ($1 = 7.1975 Chinese yuan). QALY quality-adjusted life-year. GDP gross domestic product. ICUR incremental cost-utility ratio.

Supplementary Figure 2. Cost-effectiveness plane and acceptability curve simulating all parameters (Scenario 3: discount rate = 8%).

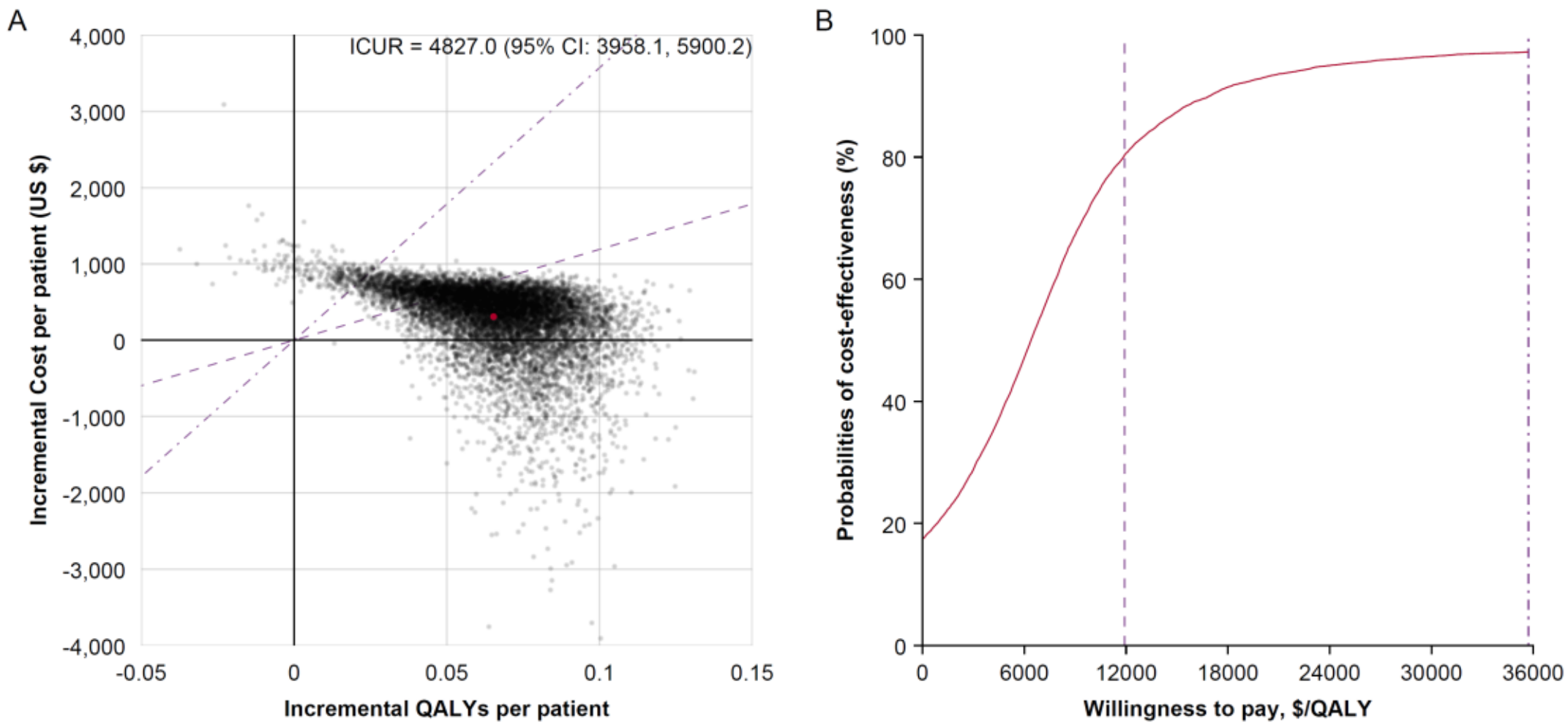

A Grey point in the cost-effectiveness plane represents one of 10,000 simulations of incremental cost and QALY pairs, varying all parameters in Supplementary Table 3. The red point indicates the base-case estimate. **B** The red acceptability curve shows the proportion of simulations deemed cost-effective across varying willingness-to-pay thresholds, indicating that telemedicine-based integrated management is cost-effective. The dashed and dot-dashed purple lines denote one-time and three-times rural China's per-capita GDP in 2024 ($11,906 and $35,718), respectively. Costs were expressed in 2024 US dollars ($1 = 7.1975 Chinese yuan). QALY quality-adjusted life-year. GDP gross domestic product. ICUR incremental cost-utility ratio.

Supplementary Figure 3. Cost-effectiveness plane and acceptability curve simulating all parameters (Scenario 4: 25 AF patients per village doctor).

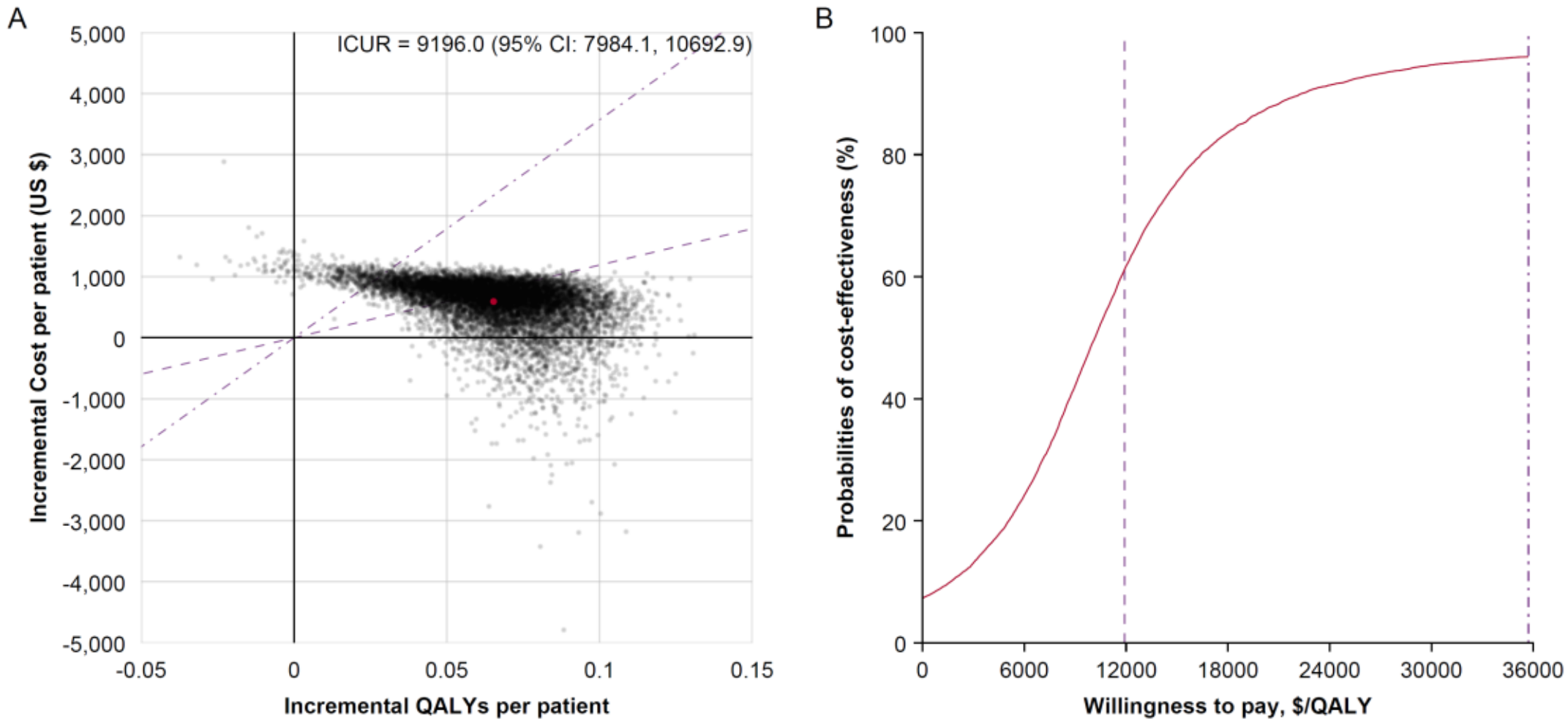

A Grey point in the cost-effectiveness plane represents one of 10,000 simulations of incremental cost and QALY pairs, varying all parameters in Supplementary Table 3. The red point indicates the base-case estimate. **B** The red acceptability curve shows the proportion of simulations deemed cost-effective across varying willingness-to-pay thresholds, indicating that telemedicine-based integrated management is cost-effective. The dashed and dot-dashed purple lines denote one-time and three-times rural China's per-capita GDP in 2024 ($11,906 and $35,718), respectively. Costs were expressed in 2024 US dollars ($1 = 7.1975 Chinese yuan). QALY quality-adjusted life-year. GDP gross domestic product. ICUR incremental cost-utility ratio.

Supplementary Figure 4. Cost-effectiveness plane and acceptability curve simulating all parameters (Scenario 5: 15 AF patients per village doctor).

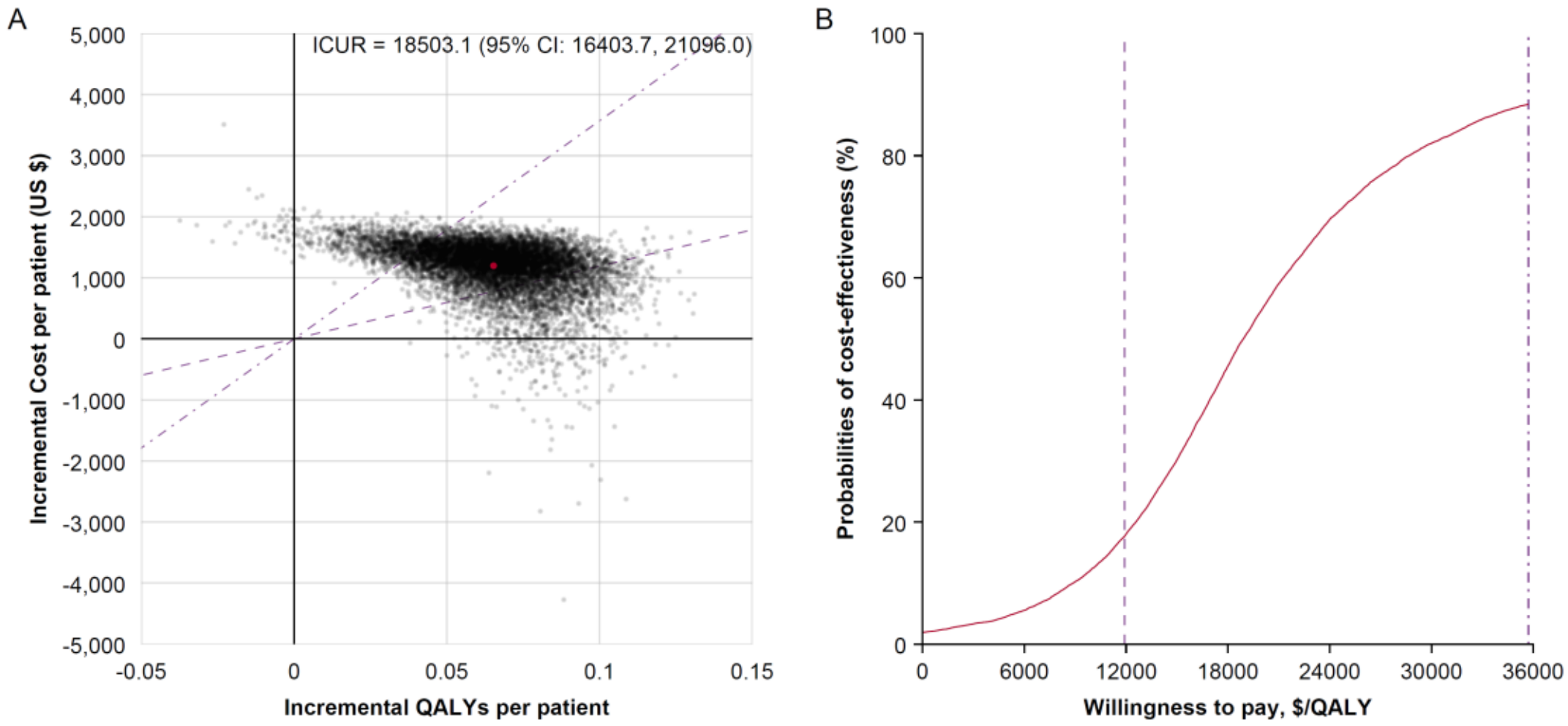

A Grey point in the cost-effectiveness plane represents one of 10,000 simulations of incremental cost and QALY pairs, varying all parameters in Supplementary Table 3. The red point indicates the base-case estimate. **B** The red acceptability curve shows the proportion of simulations deemed cost-effective across varying willingness-to-pay thresholds, indicating that telemedicine-based integrated management is cost-effective. The dashed and dot-dashed purple lines denote one-time and three-times rural China's per-capita GDP in 2024 ($11,906 and $35,718), respectively. Costs were expressed in 2024 US dollars ($1 = 7.1975 Chinese yuan). QALY quality-adjusted life-year. GDP gross domestic product. ICUR incremental cost-utility ratio.

Supplementary Figure 5. Cost-effectiveness plane and acceptability curve simulating all parameters (Scenario 6: 10 AF patients per village doctor).

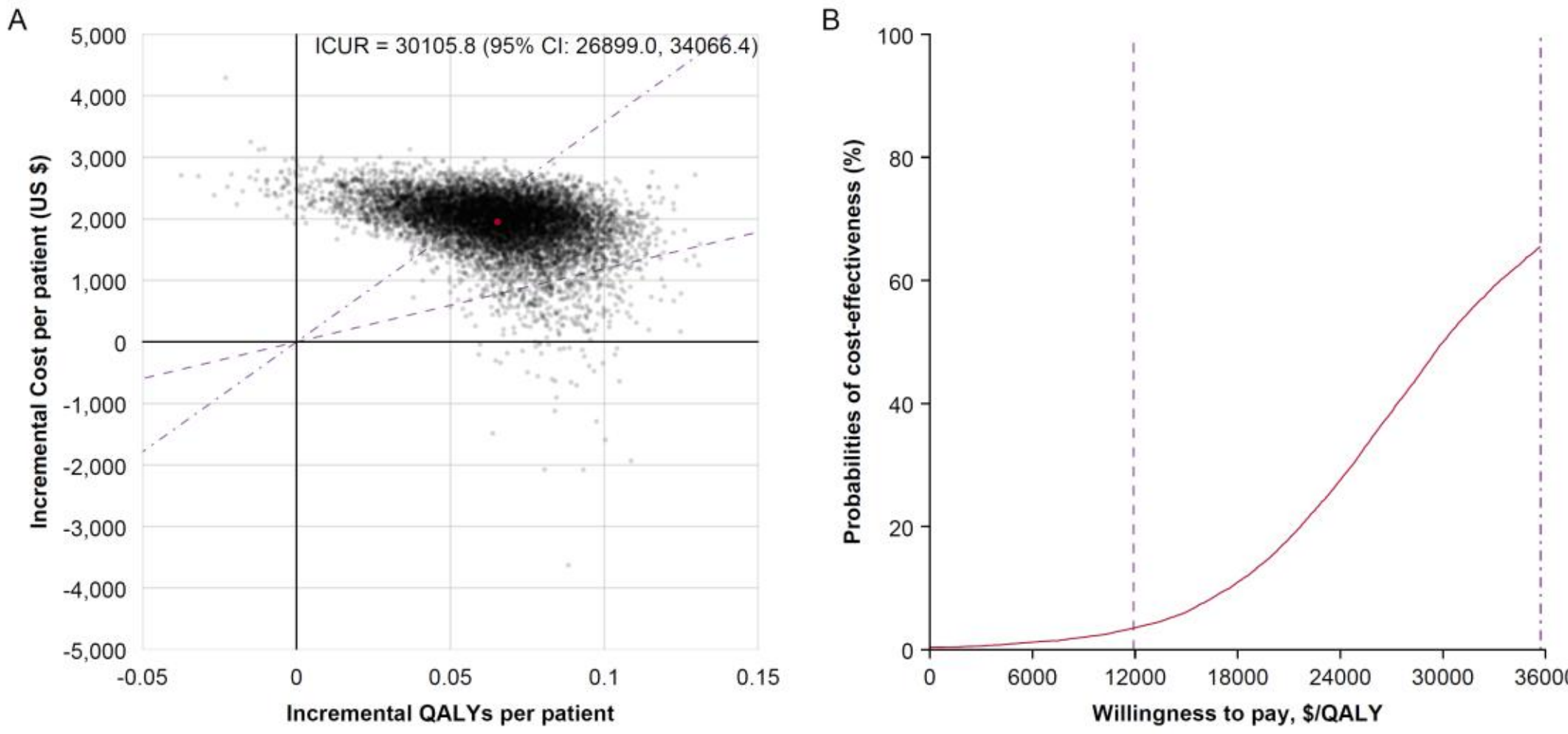

A Grey point in the cost-effectiveness plane represents one of 10,000 simulations of incremental cost and QALY pairs, varying all parameters in Supplementary Table 3. The red point indicates the base-case estimate. **B** The red acceptability curve shows the proportion of simulations deemed cost-effective across varying willingness-to-pay thresholds, indicating that telemedicine-based integrated management is cost-effective. The dashed and dot-dashed purple lines denote one-time and three-times rural China's per-capita GDP in 2024 ($11,906 and $35,718), respectively. Costs were expressed in 2024 US dollars ($1 = 7.1975 Chinese yuan). QALY quality-adjusted life-year. GDP gross domestic product. ICUR incremental cost-utility ratio.

References:

Chang, J., Deng, Q., Guo, M., Ezzati, M., Baumgartner, J., Bixby, H., Chan, Q., Zhao, D., Lu, F., Hu, P., & others. (2021). Trends and inequalities in the incidence of acute myocardial infarction among Beijing townships, 2007–2018. *International Journal of Environmental Research and Public Health*, *18*(23), 12276. https://doi.org/10.3390/ijerph182312276

Chen, Y., Wright, N., Guo, Y., Turnbull, I., Kartsonaki, C., Yang, L., Bian, Z., Pei, P., Pan, D., Zhang, Y., & others. (2020). Mortality and recurrent vascular events after first incident stroke: a 9-year community-based study of 0· 5 million Chinese adults. *The Lancet Global Health*, *8*(4), e580–e590. https://doi.org/10.1016/S2214-109X(20)30069-3

Chu, M., Zhang, S., Gong, J., Yang, S., Yang, G., Sun, X., Wu, D., Xia, Y., Jiao, J., Peng, X., Peng, Z., Hong, L., Wang, Z., Li, M., Lip, G. Y. H., Chen, M., Guo, Y., Liu, X., Lu, S., & on behalf of the MIRACLE-AF Investigators. (2025). Telemedicine-based integrated management of atrial fibrillation in village clinics: a cluster randomized trial. *Nature Medicine*, *31*(4), 1276–1285. https://doi.org/10.1038/s41591-025-03511-2

Gresham, G. E., Kelly-Hayes, M., Wolf, P. A., Beiser, A. S., Kase, C. S., & D'Agostino, R. B. (1998). Survival and functional status 20 or more years after first stroke: the Framingham Study. *Stroke*, *29*(4), 793–797. https://doi.org/10.1161/01.STR.29.4.793

He, J., Ouyang, N., Guo, X., Sun, G., Li, Z., Mu, J., Wang, D. W., Qiao, L., Xing, L., Ren, G., & others. (2023). Effectiveness of a non-physician community health-care provider-

led intensive blood pressure intervention versus usual care on cardiovascular disease (CRHCP): an open-label, blinded-endpoint, cluster-randomised trial. *The Lancet*, *401*(10380), 928–938. https://doi.org/10.1016/S0140-6736(22)02603-4

King, J. B., Shah, R. U., Bress, A. P., Nelson, R. E., & Bellows, B. K. (2016). Cost-effectiveness of sacubitril-valsartan combination therapy compared with enalapril for the treatment of heart failure with reduced ejection fraction. *JACC: Heart Failure*, *4*(5), 392–402. https://doi.org/10.1016/j.jchf.2016.02.007

Kotalczyk, A., Guo, Y., Stefil, M., Wang, Y., Lip, G. Y. H., & Investigators, the C. R. (2022). Effects of the Atrial Fibrillation Better Care Pathway on Outcomes Among Clinically Complex Chinese Patients With Atrial Fibrillation With Multimorbidity and Polypharmacy: A Report From the ChiOTEAF Registry. *Journal of the American Heart Association*, *11*(7), e024319. https://doi.org/10.1161/JAHA.121.024319

Levin, L.-Å., Wallentin, L., Bernfort, L., Andersson, D., Storey, R. F., Bergström, G., Lamm, C.-J., Janzon, M., & Kaul, P. (2013). Health-related quality of life of ticagrelor versus clopidogrel in patients with acute coronary syndromes—results from the PLATO trial. *Value in Health*, *16*(4), 574–580. https://doi.org/10.1016/j.jval.2013.01.013

Lv, W., Wang, A., Wang, Q., Wang, R., Xu, Q., Wu, S., Han, Y., Jiang, Y., Lin, J., Jing, J., & others. (2024). One-year direct and indirect costs of ischaemic stroke in China. *Stroke and Vascular Neurology*, *9*(4). https://doi.org/10.1136/svn-2023-002296

National Bureau of Statistics of China. (2021). *China Population Census Yearbook 2020*. Beijing: China Statistics Press.

National Health Commission. (2022). *China Health Statistical Yearbook 2022*. Beijing: China

Union Medical College Press.

Song, X., Lan, L., Zhou, T., Yin, J., & Meng, Q. (2021). Economic Burden of Major Diseases in China in 2013. *Frontiers in Public Health*, *9*. https://doi.org/10.3389/fpubh.2021.649624

Tu, W.-J., & Wang, L.-D. (2023). China stroke surveillance report 2021. *Military Medical Research*, *10*(1), 33. https://doi.org/10.1186/s40779-023-00463-x

Tu, W.-J., Zhao, Z., Yin, P., Cao, L., Zeng, J., Chen, H., Fan, D., Fang, Q., Gao, P., Gu, Y., & others. (2023). Estimated burden of stroke in China in 2020. *JAMA Network Open*, *6*(3), e231455–e231455. https://doi.org/10.1001/jamanetworkopen.2023.1455

Wang, H., Chai, K., Du, M., Wang, S., Cai, J.-P., Li, Y., Zeng, P., Zhu, W., Zhan, S., & Yang, J. (2021). Prevalence and incidence of heart failure among urban patients in China: a national population-based analysis. *Circulation: Heart Failure*, *14*(10), e008406. https://doi.org/10.1161/CIRCHEARTFAILURE.121.008406

Wang, K., Li, H., Kwong, W. J., Antman, E. M., Ruff, C. T., Giugliano, R. P., Cohen, D. J., Magnuson, E. A., & Investigators, E. A.-T. 48 T. (2017). Impact of spontaneous extracranial bleeding events on health state utility in patients with atrial fibrillation: results from the ENGAGE AF-TIMI 48 Trial. *Journal of the American Heart Association*, *6*(8), e006703. https://doi.org/10.1161/JAHA.117.006703

Wang, X., Moullaali, T. J., Li, Q., Berge, E., Robinson, T. G., Lindley, R., Zheng, D., Delcourt, C., Arima, H., Song, L., & others. (2020). Utility-weighted modified Rankin scale scores for the assessment of stroke outcome: pooled analysis of 20 000+ patients. *Stroke*, *51*(8), 2411–2417.

https://doi.org/10.1161/STROKEAHA.119.028523

Yang, X., Geng, T., Peng, Y., Cui, L., Chen, S., Wang, G., Gao, X., & Wu, S. (2024). Associations between cardiac arrhythmias and cardiovascular disease incidence and all-cause mortality: the Kailuan study. *BMC Public Health*, *24*(1), 3266. https://doi.org/10.1186/s12889-024-20703-6

Ye M., Li J., Lu E., Tan Z., & Cui F. (2025). Analyzing the Current Situation of Telemedicine Construction and Operation in Secondary Hospitals in China. *Chinese Health Service Management*, *42*(4), 392–397.

Zhang, W., Yi, C., Lei-Xiao, H., Jia-Hui, X., Xiao-Fei, Y., Wen-Yuan-Yue, W., Xin-Yu, W., Xiang, Q.-Y., Qin, T., Xiao-Long, W., & others. (2025). Exploring urban versus rural disparities in atrial fibrillation: prevalence and management trends among elderly Chinese in a screening study. *Journal of Geriatric Cardiology: JGC*, *22*(2), 246. https://doi.org/10.26599/1671-5411.2025.02.001
